# Driven to the edge: Species distribution modeling of a Clawed Salamander (Hynobiidae: *Onychodactylus koreanus*) predicts range shifts and drastic decrease of suitable habitats in response to climate change

**DOI:** 10.1002/ece3.8155

**Published:** 2021-10-01

**Authors:** Yucheol Shin, Mi‐Sook Min, Amaël Borzée

**Affiliations:** ^1^ Laboratory of Animal Behaviour and Conservation College of Biology and the Environment Nanjing Forestry University Nanjing China; ^2^ Department of Biological Sciences College of Natural Science Kangwon National University Chuncheon Korea; ^3^ Research Institute for Veterinary Science College of Veterinary Medicine Seoul National University Seoul Korea

**Keywords:** amphibian, climate change, Korean Peninsula, *Onychodactylus*, Salamander

## Abstract

Climate change is one of the major threats to global amphibian diversity, and consequently, the species distribution is expected to shift considerably in the future. Therefore, predicting such shifts is important to guide conservation and management plans. Here, we used eight independent environmental variables and four representative concentration pathways (RCPs) to model the current and future habitat suitability of the Korean clawed salamander (*Onychodactylus koreanus*) and then defined the dispersal limits of the species using cost distance analysis. The current habitat suitability model generated using the maximum entropy algorithm was highly consistent with the known distribution of the species and had good predictive performance. Projections onto years 2050 and 2070 predicted a drastic decrease of habitat suitability across all RCPs, with up to 90.1% decrease of suitable area and 98.0% decrease of optimal area predicted from binary presence grids. The models also predicted a northeastward shift of habitat suitability toward high‐elevation areas and a persistence of suitability along the central ridge of the Baekdudaegan Range. This area is likely to become a climatic refugium for the species in the future, and it should be considered as an area of conservation priority. Therefore, we urge further ecological studies and population monitoring to be conducted across the range of *O. koreanus*. The vulnerability to rapid climate change is also shared by other congeneric species, and assessing the impacts of climate change on these other species is needed to better conserve this unique lineage of salamanders.

## Graphical abstract

Maximum entropy models of current habitat suitability for the Korean clawed salamander were highly consistent with known distribution and had good predictive performance. Projections onto years 2050 and 2070 predicted northeastward habitat suitability shifts into higher elevation areas and a drastic decrease in both suitable and optimal area in response to climate change.

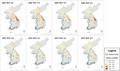

## 1 INTRODUCTION

Climate change accelerated by anthropogenic activities is having profound effects on the distribution of global biodiversity. With rapidly changing climate, faunal distributions are expected to shift across latitudinal and elevational gradients (Chen et al., 2011; Enriquez‐Urzelai et al., 2020; Parmesan & Yohe, 2003; Smeraldo et al., 2021; Wilson et al., 2007). Although such range shifts can lead to range expansions and population increase in some species (Borzée, Andersen, et al., 2019; Hu et al., 2020), many species are likely to experience range contractions and displacement from their suitable habitats, ultimately leading to population decline and extinction (Ashrafzadeh et al., 2019; Borzée, Andersen, et al., 2019; Hu et al., 2020; Mothes et al., 2020; Zhang, Mammola, et al., 2020).

Among vertebrates, amphibians are especially prone to the detrimental effects of climate change. Their ecology is closely tied to requirements for humidity and temperature for cutaneous respiration (Carey & Alexander, 2003), and many species undergo biphasic life histories with an aquatic stage at least during their larval phase (Duellman & Trueb, 1994). These aquatic ecological traits and related habitat requirements can be easily disturbed by rapid climate change (Bickford et al., 2010). With 40% of all amphibian species already threatened (IUCN, 2020), the effects of climate change are likely to exacerbate the problems caused by other known threats including habitat destruction, infectious diseases, overexploitation, and pollution (Beebee & Griffiths, 2005; Gallant et al., 2007; Lips, 1998; Lips et al., 2006; Rowly et al., 2016; Sodhi et al., 2008; Wake, 2012). Therefore, predicting range shifts as a consequence of climate change is important to better guide management, policy‐making, and conservation decisions, as well as to determine important areas for conservation.

Correlative species distribution models (SDMs) have become the main method for conducting such predictions (Elith & Leathwick, 2009). Generated by correlating species' distribution and a set of environmental predictors, SDMs provide a representation of continuous habitat suitability across the landscape (Elith & Leathwick, 2009; Guisan & Zimmermann, 2000). The SDMs can then be projected onto different space to locate suitable habitats outside the species' known distributions (Pyron et al., 2008; Raffini et al., 2020; Srivastava et al., 2021), as well as to different time periods to predict range shifts under climate change (Borzée, Andersen, et al., 2019; Hu et al., 2020; Mothes et al., 2020). The development of user‐friendly interfaces and statistical packages such as the software MaxEnt have greatly enhanced the ability to implement such projections (Phillips et al., 2017), opening the doors for this method to be applied to other species with presumed vulnerability to climate change.

**FIGURE 1:**
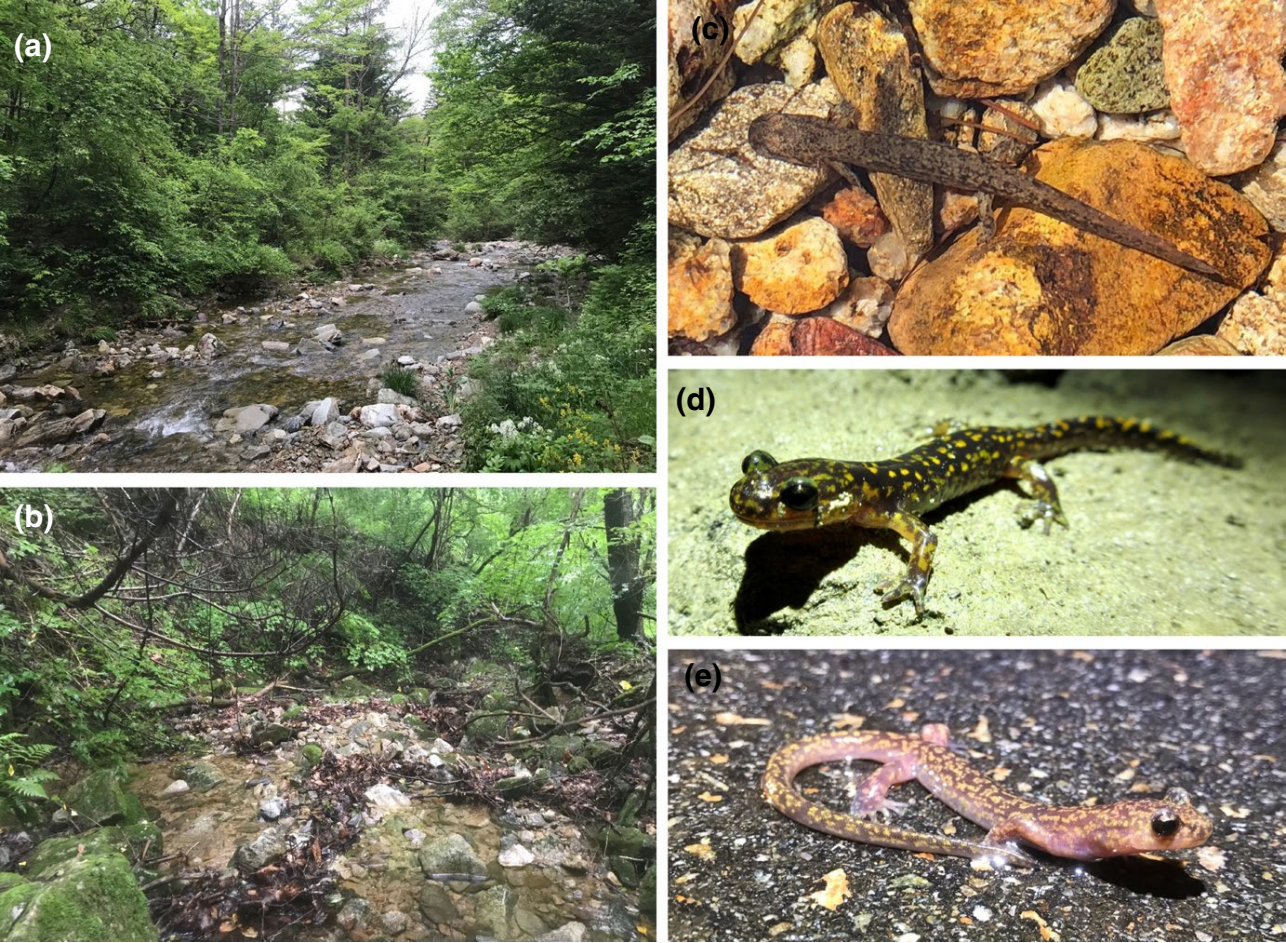
The habitats and various life‐history stages of *Onychodactylus koreanus*. Typical mountainous stream environments suitable to *O. koreanus* in Hongcheon (a) and Yanggu (b), Republic of Korea. (c) *O. koreanus* has an extended aquatic larval stage and the larvae are highly dependent on clean and low‐temperature streams with high levels of dissolved oxygen. Photographed in the same area as (b). (d) Subadult *O. koreanus* observed in Chuncheon, Republic of Korea. (e) Adult *O. koreanus* observed in Hwacheon, Republic of Korea. Adult *O. koreanus* typically inhabits moist forest floor and reproduce in subterranean environments. All photographs were taken in 2021 by YS

Here, we applied correlative species distribution modeling to estimate current and future habitat suitability of the Korean clawed salamander (*Onychodactylus koreanus*), a species of hynobiid salamander endemic to the Korean Peninsula (Poyarkov et al., 2012; Lee & Park, 2016). This species is primarily found in the mountainous regions of the Korean Peninsula, mainly along the mountains branching from the Baekdudaegan Range, in close association with clear low‐temperature streams and forests (Figure 1). Due to their characteristics and ecology, members of the genus *Onychodactylus* are highly likely to become threatened by climate change (Suk et al., 2018), similarly to other Korean salamanders (Borzée, Andersen, et al., 2019). First, unlike other hynobiids, the members of this genus are lungless, making them more stringently bound to moist and cool environments (Kuzmin, 1995; Poyarkov et al., 2012). Second, unlike most plethodontids, a diverse family of lungless salamanders, *Onychodactylus* goes through aquatic reproduction and larval phase (Park, 2005). This requires unpolluted and low‐temperature mountainous or subterranean water bodies with high levels of dissolved oxygen (Hong, 2017; Jung, 2020; Kuzmin, 1995; Lee & Park, 2016), environments that are prone to degradation by climate change. Previous studies on plethodontids have predicted range shifts and the loss of diversity hotspots due to climate change (Borzée, Andersen, et al., 2019; Milanovich et al., 2010; Parra‐Olea et al., 2005), and similar patterns are expected for *Onychodactylus*. However, while the detrimental effects of climate change on the genus have been speculated (Suk et al., 2018), no study has applied SDMs to investigate potential range shifts and future habitat suitability in response to climate change to our knowledge.

The effects of climate change on the distribution of *O. koreanus* will also have conservation implications for the future. In the Republic of Korea (R. Korea hereafter), the Ministry of Environment provides different levels of legal protection for herpetofauna. These legal measures include (from lowest to highest levels of protection): capture ban without permit, Endangered (category II), and Endangered (category I). While *O. koreanus* had been listed as Endangered in R Korea until 1998, the species has been delisted from this category (Hong, 2017; NIBR, 2021) and is now listed as Least Concern (LC) under a national threat assessment (NIBR, 2019), although permits are required to collect specimens. However, the lowering of conservation status in R Korea is a reflection of increased sampling and subsequent discoveries of additional habitats and populations, rather than true “recovery” of the species from threats. In addition, the species is not listed under specific conservation programs in the Democratic People's Republic of Korea (D.P.R. Korea hereafter). Conservation requirements are however expected to change with rapid climate change, and the protection status of the species will require appropriate adjustments accordingly.

Therefore, the goals of this study are to (a) estimate current habitat suitability of *O. koreanus*, (b) assess the potential impact of climate change on the future distribution of the species, and (c) provide baseline information for future conservation efforts on the species. Considering the known ecological characteristics of *O. koreanus*, we hypothesize that climate change will have adverse effects on the future distribution of this species by drastically reducing habitat suitability.

## 2 MATERIALS AND METHODS

### 2.1 Occurrence records

**FIGURE 2:**
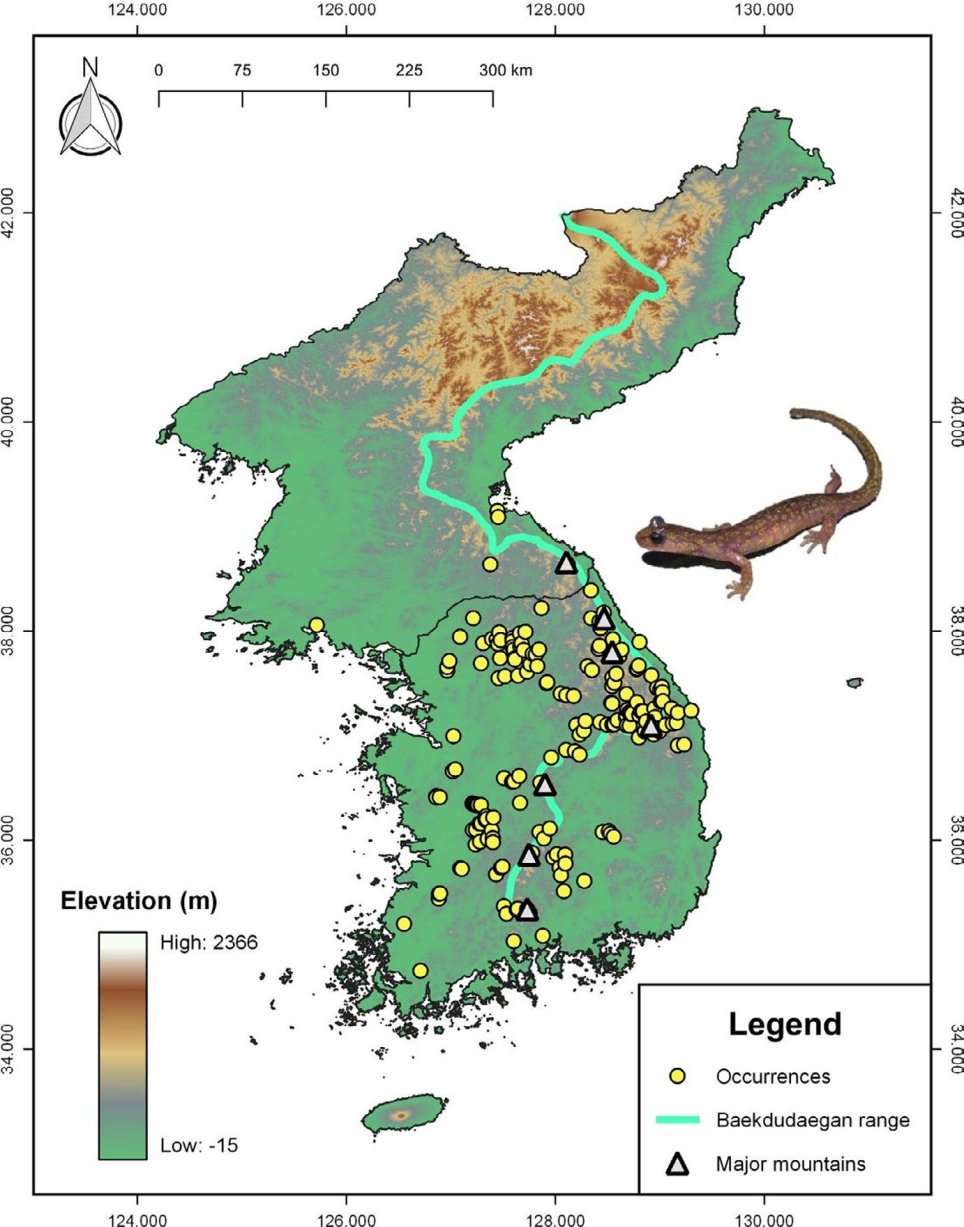
Distribution of occurrence points of *Onychodactylus koreanus* on the Korean Peninsula used for species distribution modeling. We compiled the occurrence records from our survey data, Global Biodiversity Information Facility (GBIF), VertNet, georeferenced vouchers catalogued in Shin, Jang, et al. (2020), occurrence dataset from Borzée, Litvinchuk, and Andersen (2021), and the results of the 4th National Ecosystem Surveys. Note the occurrence points are mostly recorded along the Baekdudaegan Range and mountains branching from Baekdudaegan. “Major mountains” (grey triangle) denote the locations of several major mountains along the Baekdudaegan Range with known or likely presence of *O. koreanus*. From top to bottom, the mountain names are as follows: Geumgang, Seorak, Odae, Taebaek, Sokri, Deokyu, and Jiri

We obtained and compiled occurrence records of *Onychodactylus koreanus* from various sources, including our own survey data, the Global Biodiversity Information Facility (GBIF; accessed 7 October 2020; https://doi.org/10.15468/dl.khx67d), VertNet (searched under *O. fischeri*; accessed 22 September 2020), georeferenced vouchers deposited in the Ewha Womans University Natural History Museum (EWNHM; Sabaj, 2019; catalogued in Shin et al., 2020), and herpetofauna survey results from the 4th National Ecosystem Survey (NES), conducted by the National Institute of Ecology between years 2014 and 2018 (available from the EcoBank platform; Kim et al., 2021; accessed 13 December 2020). We visually inspected all public database records provided with photographs to ensure correct species identification. Furthermore, we incorporated georeferenced data points from the D.P.R. Korea to improve model estimation. This dataset is available from the Mendeley Data repository (https://doi.org/10.17632/z7kgyy8chp.1; Borzée et al., 2021; Borzée, Litvinchuk, Ri, et al., 2021). Because of the presence of other *Onychodactylus* species in D.P.R. Korea (especially *O. zhaoermii* and *O. zhangyapingi*; Poyarkov et al., 2012; Borzée, Litvinchuk, Ri, et al., 2021), we initially filtered this dataset to exclude data points near the border between D.P.R. Korea and the People's Republic of China (hereafter China). Furthermore, we excluded data points of “*O. koreanus*” recorded around southeastern R. Korea (“Clade E” of Poyarkov et al., 2012), from the dataset due to the species‐level degree of genetic divergence of this population to other populations of *O. koreanus* (Poyarkov et al., 2012). This resulted in a total of 922 data points including numerous locations throughout R. Korea and several locations in D.P.R. Korea, collected from 1911 to 2020 (https://doi.org/10.17632/yc3nw4d9f2.2; Shin et al., 2021; Figure 2). While the occurrence dataset is naturally correlated temporally due to ecological characteristics of the species (e.g., cryptic amphibian with increased activity and detection during rainy months), the risk of temporal correlation leading to biased occurrence sampling is unlikely in our case. This is because our target species is a strict habitat specialist that occupies narrow environments along mountain streams and forests immediately adjacent to them, such that the observation of larvae in a given area is a good indicator of the presence of adults in the same area, and vice versa (Hong, 2017; Lee & Park, 2016). Therefore, accounting for spatial sampling bias was more important in our case than considering temporal bias, and we accounted for spatial sampling bias by spatial occurrence thinning and modified background selection (see *Model preparation* section below).

### 2.2 Model preparation

We initially considered a total of 27 environmental variables to model the current habitat suitability of *O. koreanus*. First, we obtained 19 bioclimatic variables from WorldClim 2.1 (https://www.worldclim.org/; Fick & Hijmans, 2017) at the 30‐arc‐second resolution. These bioclimatic variables are derived from climate data averaged across the years between 1970 and 2000, thereby creating temporal mismatch between the occurrence and climate data. However, we consider that such mismatch does not hinder the model predictions as our target organism is a slow‐dispersing species. Furthermore, as sampling efforts for the species are uneven between the years 1911 and 2020, strictly enforcing temporal match between occurrence and climate data would mean that only a small subset of occurrence points could be used for modeling. As this can lead to biased model estimates, we consider the use of the full occurrence dataset to be more beneficial than enforcing temporal match between data, while acknowledging the presence of the temporal mismatch.

In addition to the bioclimatic variables, we included two topographic variables (slope and elevation), three forest cover variables (needleleaf forest cover, broadleaf forest cover, and mixed/other trees cover), two variables representing human activities (urban landscape cover and human footprint), and a raster representing the distance to the nearest water bodies, as these variables are highly relevant to the ecology of *O. koreanus* (Kuzmin, 1995; Poyarkov et al., 2012; Lee & Park, 2016). We downloaded the elevation layer from WorldClim 2.1, slope and land‐cover layers from EarthEnv (https://www.earthenv.org; Amatulli et al., 2018; Tuanmu & Jetz, 2014), and the human footprint layer from the NASA Socioeconomic Data and Applications Center (Last of the Wild version 3; Venter et al., 2018). The distance to water bodies was calculated as a Euclidean distance from water bodies derived from the elevation layer in ArcMap 10.8.1 (ESRI, Redlands, CA). All nonclimatic variables were also downloaded or generated at the 30‐arc‐second resolution.

As a model calibration area, we used the entire Korean Peninsula to encompass the full extent of distribution of *O. koreanus*, while acknowledging the uncertain northern range limits of the species (see *Estimation of dispersal limits* section below). We processed our layers by masking them with the boundaries of the Korean Peninsula in the software R (version 4.1; R Core Team, 2021) using the packages *raster* (Hijmans, 2019) and *rgdal* (Bivand et al., 2021).

Maximum entropy (implemented in the software MaxEnt) is a robust method of species distribution modeling with presence‐only data (Phillips et al., 2017). However, one of the major issues with this type of data is sampling bias (Kramer‐Schadt et al., 2013; Phillips et al., 2009). As a presence‐only algorithm, MaxEnt requires a set of background points for model construction (Merow et al., 2013). By default, 10,000 background points are randomly selected across the entire extent of the study, but this does not take sampling bias into account (Phillips et al., 2009). As our occurrence data had both regional (for GBIF and NES data) and national (between R. Korea and D.P.R. Korea) biases (Syfert et al., 2013), we employed target group background sampling (Merow et al., 2013; Phillips et al., 2009). To do so, we first extracted all Korean amphibian data points from GBIF, Borzée, Litvinchuk, and Andersen (2021), and the 4th National Ecosystem Surveys, resulting in a total of 20,997 occurrences (https://doi.org/10.17632/yc3nw4d9f2.2; Shin, Min, et al., 2021). After spatially thinning this dataset using a 1‐km distance parameter in the R package *spThin* (Aiello‐Lammens et al., 2015), 4,523 unique data points remained (https://doi.org/10.17632/yc3nw4d9f2.2; Shin, Min, et al., 2021). We converted these data points into a density raster reflecting sampling intensity across the Korean Peninsula and used this raster for bias correction in subsequent MaxEnt modeling.

To reduce the clustering of occurrence points, we spatially thinned our occurrence data so that each data point is at least one kilometer apart, thereby putting only one occurrence point within each 30‐arc‐second (~1 km) grid cell of environmental layers. This procedure was conducted in R using the package *spThin* (Aiello‐Lammens et al., 2015) and reduced the number of data points from 922 to 187 occurrence data used to calibrate the models (https://doi.org/10.17632/yc3nw4d9f2.2; Shin, Min, et al., 2021). After reducing spatial clustering, the occurrence points still encompassed the totality of the known range of *O. koreanus*. Also, considering the known capability of MaxEnt algorithm to predict habitat suitability even with small sample sizes (Pearson et al., 2007), our spatially thinned occurrence dataset is sufficiently large to generate models with good predictive ability.

We used a two‐step approach to select environmental variables to calibrate our models. First, we conducted five bootstrap replicated runs in MaxEnt version 3.4.1 (https://biodiversityinformatics.amnh.org/open_source/maxent/) with the spatially thinned occurrence dataset, 27 environmental layers, auto features, and a default regularization multiplier (= 1). For background point selection, we used the density raster to sample 10,000 target group background points. From this initial run, we retained nine variables with contribution scores greater than 3%. To reduce multicollinearity among the retained variables, we then conducted a Spearman's correlation test in the R package *ntbox* (Osorio‐Olvera et al., 2020) and removed highly correlated variables if *|* coefficient *|* > 0.7. This resulted in the following set of eight environmental variables used to estimate the current habitat suitability of *O. koreanus*: bio1 (annual mean temperature), bio3 (isothermality), bio4 (temperature seasonality), bio13 (precipitation of wettest month), bio14 (precipitation of driest month), slope, needleleaf forest cover, and mixed/other trees cover. As *O. koreanus* is a lungless ectotherm living in and around mountain streams, its distribution is expected to be strongly governed by precipitation, humidity, temperature, and vegetation cover. Therefore, we considered the selection of the eight environmental variables to be appropriate. While human‐related variables were eventually excluded from the model calibration due to the initial filtering criterion, the effects of human activities can still be captured by other environmental variables (e.g., low forest cover in highly populated area). Nevertheless, we included human‐related variables to calculate cost rasters for the estimation of dispersal limits as human activities are still important to determine the species' distribution and dispersal limits (see *Estimation of dispersal limits* section below).

To optimize model parameters for calibration, we tested a combination of six MaxEnt feature classes (Linear, Linear Quadratic, Hinge, Linear Quadratic Hinge, Linear Quadratic Hinge Product, and Linear Quadratic Hinge Product Threshold), and 16 regularization multipliers (ranging from 0.5 to 8 with a 0.5 increment) under two cross‐validation schemes: 10‐fold cross‐validation and spatial block cross‐validation. While the former method is commonly used to generate and test SDMs, we also tested the latter method as its use has been recommended when model transfer to different environmental conditions is the goal (Muscarella et al., 2014; Kass et al., 2021). We used 10,000 target group background points to generate candidate models under each cross‐validation method. To select optimal model parameters from the sets of candidate models, we applied and compared two selection criteria. First, we selected the model with a combination of feature classes and regularization multiplier that had the lowest AICc value, describing model fit and complexity (Warren & Seifert, 2011; Muscarella et al., 2014). Second, we selected the model that had the lowest 10% omission rate as well as the highest test AUC (sequential selection method; Kass et al., 2021). For both cross‐validation methods, the optimal parameters based on AICc were the LQHPT features combined with a regularization multiplier of 1.5. On the other hand, based on the sequential selection method, the optimal parameters under the spatial block cross‐validation was the L feature with a regularization multiplier of 4.5, while the optimal parameters under 10‐fold cross‐validation were the H feature combined with a regularization multiplier of 3.5. To select the final optimal model parameters, we projected the habitat suitability predicted from these three parameters across the landscape. As the northern distribution limit of *O. koreanus* in D.P.R. Korea remains uncertain due to the presence of closely related species in the country (Poyarkov et al., 2012; Borzée, Litvinchuk, Ri, et al., 2021), selecting model parameters that predicted habitat suitability in D.P.R. Korea with minimum overprediction was key to generate accurate current habitat suitability model. According to these criteria, we selected the model parameters with the lowest AICc (LQHPT 1.5) to calibrate the final current habitat suitability model. We conducted the model tuning procedure using R packages *dismo* (Hijmans et al., 2017) and *ENMeval* (Muscarella et al., 2014; Kass et al., 2021).

### 2.3 Current habitat suitability

We used MaxEnt for our modeling because of the algorithm's ability to explicitly account for spatial bias in the occurrence dataset (via a density raster; Shin, Messenger, et al., 2021). We calibrated the current habitat suitability model with 10,000 target group background points and 10‐fold cross‐validation (Thapa et al., 2018). We applied all MaxEnt feature classes (LQHPT) with a regularization multiplier of 1.5, as determined by the *ENMeval* run. We selected cloglog as the output format (Borzée, Andersen, et al., 2019; Di Pasquale et al., 2020; McDonald et al., 2021; Phillips et al., 2017), and we took the average of the 10 replicated models for the final model evaluation. We also used a jackknife analysis to assess the contribution of each variable to the model.

We evaluated model performances using both threshold‐independent (area under the receiver operating characteristic curve; AUC) and threshold‐dependent (True Skill Statistic; TSS; Allouche et al., 2006) metrics. To calculate TSS, we used the maximum training sensitivity plus specificity threshold (= 0.5175). We also evaluated the degree of model overfitting using a threshold‐independent metric: AUC_DIFF_, computing the difference between model training AUC and model testing AUC, for which values close to 0 indicate little overfitting of models (Warren & Seifert, 2011). We additionally evaluated the model based on the known distribution of *O. koreanus* across the Korean Peninsula (Kim, 2009; Jang & Seo, 2010; Poyarkov et al., 2012; NIBR, 2019).

### 2.4 Future projections

For future projections we used the Community Climate System Model 4.0 (CCSM4) scenario downscaled from Coupled Model Intercomparison Project Phase 5 (CMIP5) data, downloaded at the 30‐arc‐second resolution from WorldClim database. This scenario has been widely applied to predict future habitat suitability of terrestrial vertebrates (Hu et al., 2020; Morovati et al., 2020; Sutton et al., 2015) and was also effective in predicting future habitat suitability of *Karsenia koreana*, another lungless salamander species endemic to the Korean Peninsula (Borzée, Andersen, et al., 2019). We implemented our projections on to two time periods: years 2050 (average for 2041–2060) and 2070 (average for 2061–2080), under four Representative Concentration Pathways (RCPs): RCP 2.6, RCP 4.5, RCP 6.0, RCP 8.5. The RCPs used herein correspond to four greenhouse gas concentration trajectories with respective radiative forcing increase of 2.6, 4.5, 6.0, and 8.5 W/m^2^ by 2100 (van Vuuren et al., 2011).

While bioclimatic variables were projected onto future conditions, we maintained topographical and land‐cover variables constant throughout all projections due to the lack of future scenarios corresponding to climate change for these variables. Although vegetation cover is likely to shift with climate change (Choi et al., 2011), thereby introducing a bias in our analyses, adding these variables was still favorable to better characterize the ecological niche of *O. koreanus* compared to using bioclimatic variables only.

### 2.5 Estimation of dispersal limits

One of the most significant limitations of SDMs is the inability of the modeling algorithms to explicitly account for the dispersal ability of organisms, and this can lead to unrealistic estimates of the current and future habitat suitability (Guisan & Thuiller, 2005; Ofori et al., 2017; Zhang, Mammola, et al., 2020). Although the dispersal ability of *O. koreanus* is unknown, it is likely to be limited as the species is a lungless ectotherm and as its distribution is tightly linked to forest environments. Thus, we tried to overcome this limitation by estimating the cost of dispersal through various landscape features (Sahlean et al., 2014).

To do so, we first generated a set cost rasters, the values of which represent the difficulty (cost) of moving through the landscape. Here, we used the following landscape and environmental features to construct the cost rasters: barren landscape cover, urban landscape cover, cultivated landscape cover, forest cover, open water bodies, elevation, human footprint, and MaxEnt habitat suitability outputs. While some of these variables were not included in habitat suitability modeling, they are nonetheless important to estimate the cost of dispersal as they provide significant barriers to dispersal (e.g., urban area). We downloaded the landscape cover and open water bodies rasters from EarthEnv (Tuanmu & Jetz, 2014), the elevation raster from WorldClim 2.1 (Fick & Hijmans, 2017), and the human footprint raster from NASA Socioeconomic Data and Applications Center (Last of the Wild version 3; Venter et al., 2018). We generated the forest cover raster by merging the three forest cover variables (needleleaf, broadleaf, and mixed/other trees; each downloaded from EarthEnv), as the effects of specific forest type on the dispersal of *O. koreanus* are unknown. All rasters followed a 30‐arc‐second (~1 km) resolution. Next, we reclassified the above rasters by manually assigning costs of dispersal to original raster values based on the ecological information on the species derived from the literature (Kuzmin, 1995; Park, 2005; Song & Lee, 2009; Poyarkov et al., 2012; Hong, 2017; Lee & Park, 2016; Jung, 2020; Shin et al., 2020) and our knowledge of the species' biology. We also relied on the literature for specific information, such as the known upper elevation limit for the species (Song & Lee, 2009). The cost values assigned to each variable are listed in detail in Appendix A. In brief, we assigned higher costs to raster values representing greater difficulties to dispersal. Areas highly permeable to dispersal (e.g., areas with high forests cover) received a cost of 1, whereas insurmountable barriers to dispersal (e.g., urban areas, lakes) received a cost of 1,000,000. Other areas with varying degrees of difficulty to dispersal received costs between 1 and 1,000,000, according to the perceived ability of the species to disperse across the landscape based on ecological knowledge on the species derived from the literature. For the cost assignment to each MaxEnt habitat suitability output model, raster values below the habitat suitability threshold (maximum training sensitivity plus specificity; MTSS) received a cost value of 1,000,000, as areas below this threshold were deemed unsuitable habitats and likewise impermeable to dispersal. The values above this threshold received decreasing costs with increasing habitat suitability (see Appendix B for the MTSS threshold value for each MaxEnt model). The resulting reclassified rasters had different numbers of cost classes (Sahlean et al., 2014). To generate the costs rasters, we merged all reclassified rasters using the Mosaic to New Raster tool in ArcMap 10.8.1., assigning the maximum cost values among overlapping cells to the output cost rasters. We generated cost rasters separately for each MaxEnt model scenario (current, 2050, and 2070, under each RCP), keeping the landscape features constant and only changing the MaxEnt habitat suitability outputs between scenarios.

Using the costs rasters and occurrence points as inputs, we conducted a cost distance analysis for each MaxEnt model scenario, using the Cost Distance tool in ArcMap 10.8.1. This analysis calculates, for each grid cell, the least accumulative cost from the source cells (occurrence points) to the edges of input cost rasters. We classified accumulative cost classes of the output cost distance rasters based on the Jenks natural breaks method at seven classes, because this method maximizes the variance between classes while minimizing the variance within classes. Then, we reclassified the cost distance rasters with the highest values of lowest cost classes as thresholds. Here, we define the reclassified cost distance rasters as dispersal limits (sensu Sahlean et al., 2014).

### 2.6 Area of suitable and optimal habitats

To calculate the total habitat area of *O. koreanus* under different climate projections through time, we converted the output models from continuous suitability maps into binary presence–absence maps. First, we cropped all model outputs with their respective dispersal limits delineated by the cost distance analyses. Next, we applied the MTSS threshold computed in the MaxEnt runs to obtain binary area of suitable habitats restricted by dispersal limits (see Appendix B for specific threshold value applied to each model scenario). We selected MTSS because this threshold maximizes correctly predicted presence as well as correctly predicted absence. It is also one of the most accurate thresholds (Dubuis et al., 2011; Liu et al., 2005; McDonald et al., 2021; Smeraldo et al., 2021). Meanwhile, binary grids generated by applying other widely used but more conservative thresholds, such as 10% omission and 0% omission (minimum presence), considerably overestimated suitable habitats in D.P.R. Korea (not shown). Due to the presence of other closely related congeners in D.P.R. Korea with uncertain distribution (Poyarkov et al., 2012; Borzée, Litvinchuk, Ri, et al., 2021), we needed to avoid overestimating the suitable habitats. The binary grids generated from conservative thresholds further overestimated the suitable habitats in R. Korea, even in areas where we know the species to be absent. Therefore, we consider our choice of threshold to be appropriate.

Next, considering that *O. koreanus* is a strict habitat specialist adapted to specific environmental conditions of mountain forests, we calculated the total area of optimal habitats within dispersal limits. Applying the approach implemented in Mothes et al. (2020), we defined an optimal habitat threshold of 0.8 to calculate the total area of optimal habitats for each model scenario. Using the area values obtained from the binary maps, we calculated the percentage of decrease in suitable and optimal areas through time across different projections. In addition, we estimated elevational shifts of future suitable and optimal areas from the elevation ranges of current suitable and optimal habitats. To do so, we extracted elevation values from both suitable and optimal binary area maps generated from current and all future projections and then conducted Mann–Whitney *U* tests between the current elevation ranges and elevation ranges of each future projection. We conducted the area calculations and Mann–Whitney *U* tests in R version 4.1 (R Core Team, 2021), and all projections, map visualizations, and associated calculations outlined herein were under the WGS 84 coordinate system.

## 3 RESULTS

### 3.1 Current habitat suitability

Based on evaluation metrics, our model is acceptable as an adequate representation of habitat suitability of *O. koreanus* (AUC = 0.866 ± 0.05 *SD*; TSS = 0.671 ± 0.01 *SD*; AUC_DIFF_ = 0.021). Among the eight environmental variables used to construct the model, slope had the highest percentage of contribution to the model, followed by annual mean temperature, precipitation of driest months, precipitation of wettest months, cover of mixed/other forests, isothermality, temperature seasonality, and needleleaf forest cover (Appendix C). Based on the response curves, the area of high suitability (>0.8) for *O. koreanus* is characterized by slope ranging from approximately 17–35°, annual mean temperature ranging from 1 to 10℃, precipitation of driest months between 27 to 45 mm, precipitation of wettest months between 300 and 400 mm, cover of mixed and other trees above 65%, isothermality ranging from approximately 17 to 28, temperature seasonality above 1,000, and needleleaf forest cover ranging from about 20% to 70% (Appendix D). Such environmental characteristics are consistent with mountainous stream and forest habitats receiving high levels of precipitation.

**FIGURE 3:**
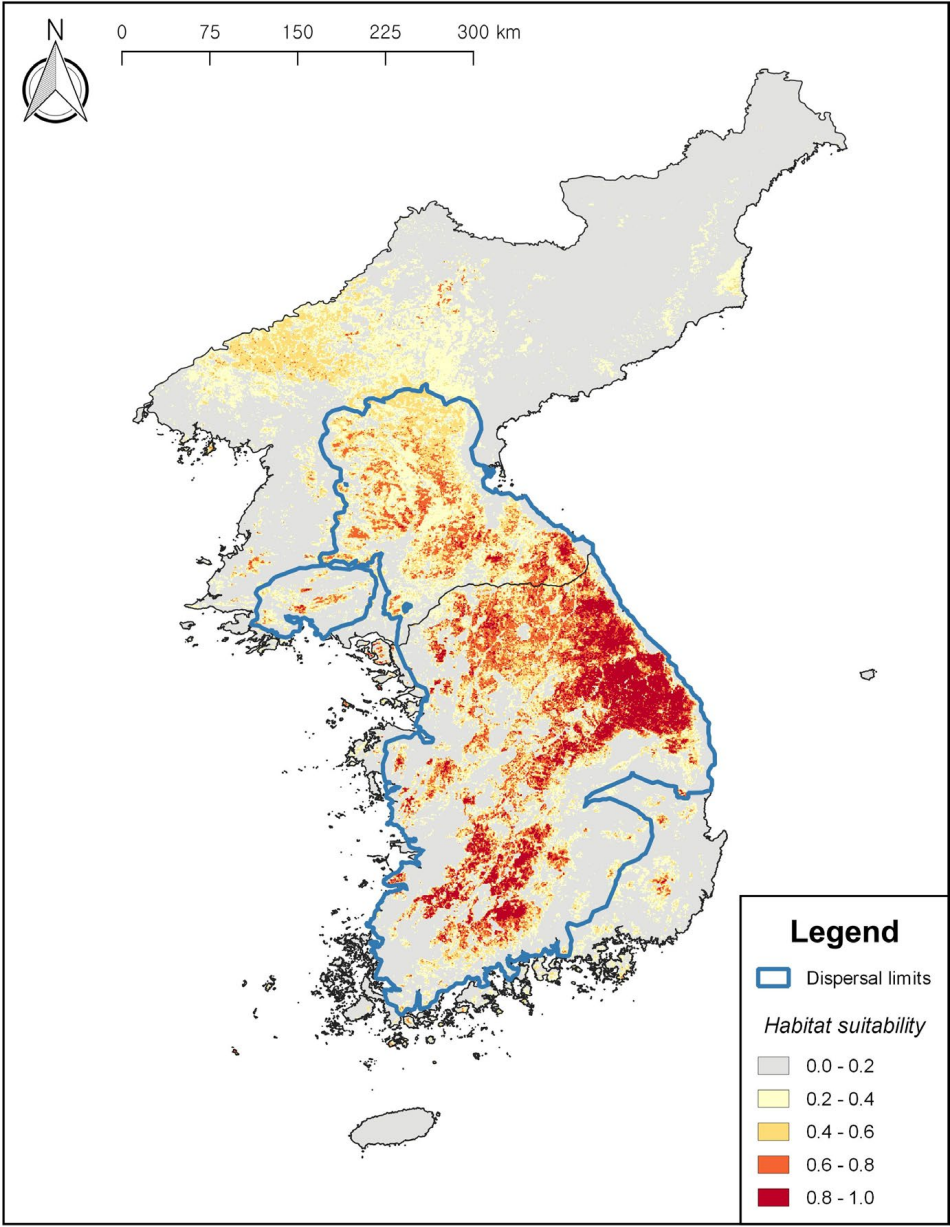
Maximum entropy model of current habitat suitability for *Onychodactylus koreanus* (AUC = 0.866 ± 0.05; TSS = 0.671 ± 0.01; AUC_DIFF_ = 0.021). The areas of high suitability (0.8–1.0) are mostly located along the Baekdudaegan Range and associated mountain chains, including mountains Geumgang, Seorak, Odae, Taebaek, Sokri, Deokyu, and Jiri. Areas of intermediate to high suitability in northern and southeastern Korean Peninsula are likely to be overestimations according to the estimation of dispersal limits based on cost distance analysis. The map scales and coordinate grids are the same as Figure 2

The current habitat suitability model was remarkably consistent with the known distribution of *O. koreanus* (for known distribution of *O. koreanus*, see Kim, 2009; Jang & Seo, 2010; Poyarkov et al., 2012; NIBR, 2019). The model highlighted areas of high suitability (>0.8; optimal area defined herein) located along the Baekdudaegan Range and associated mountains (Figure 3). This is a major mountain range that includes Geumgang, Seorak, Odae, Taebaek, Sokri, Deokyu, and Jiri mountains, where *O. koreanus* is currently known to occur or likely to be found (Poyarkov et al., 2012; NIBR, 2019; Figure 2).

In R. Korea, the eastern regions had the widest area of suitable habitats. This region is characterized by high‐elevation mountains along the Baekdudaegan Range. In contrast, a broad expanse of low suitability and isolated area of intermediate to high suitability were notable across southeastern R. Korea. This is likely to be closely related to the topography of the region, where mountainous regions bordering the main ridge of the Baekdudaegan Range transition into low‐elevation basins (Figure 2). In D.P.R. Korea, the areas of high suitability were also located along the Baekdudaegan Range and associated mountain systems with a transition from high‐to‐low habitat suitability occurring around the central region of the nation.

### 3.2 Future projections

**FIGURE 4:**
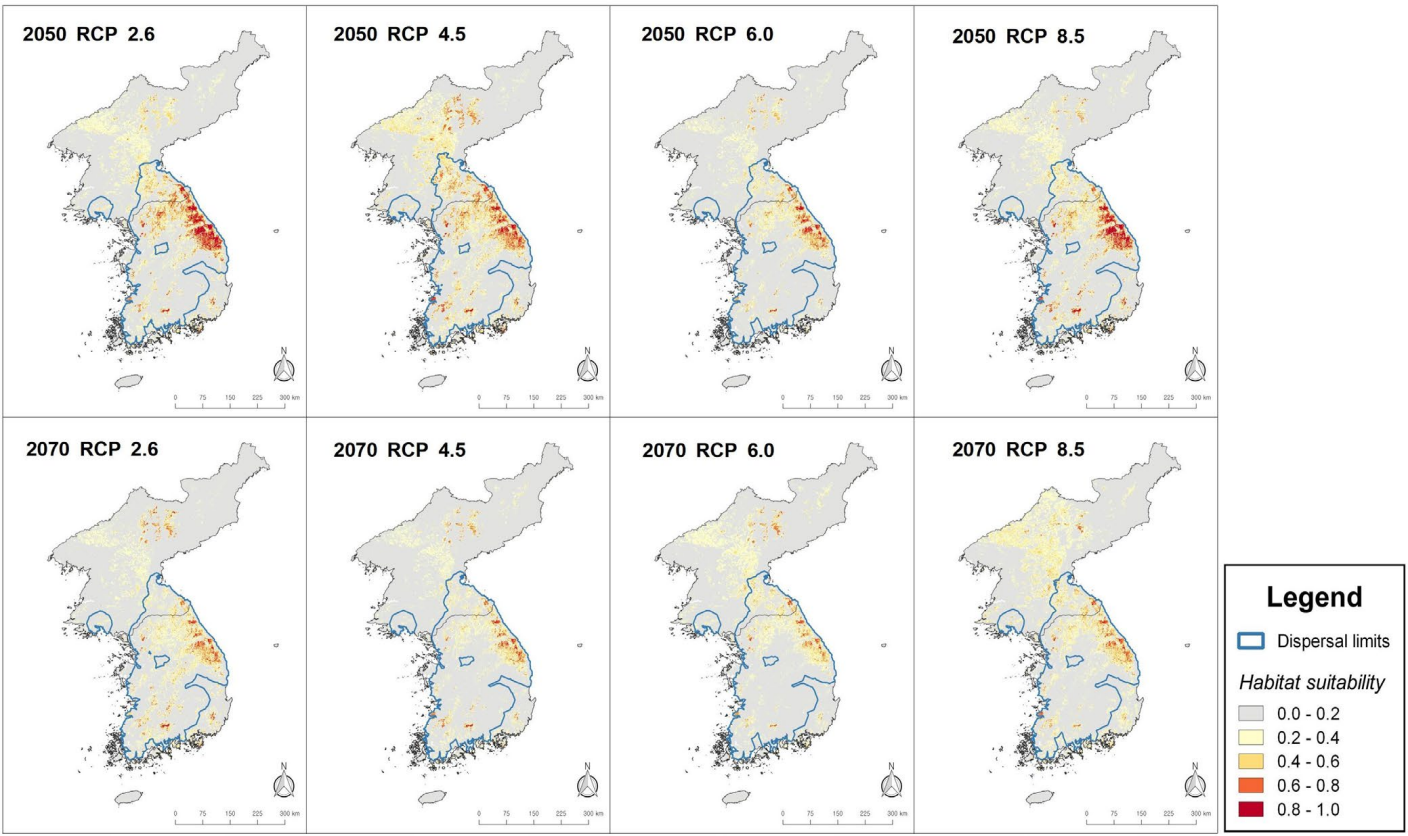
Future habitat suitability projections for *Onychodactylus koreanus* to years 2050 and 2070. Modeled under four Representative Concentration Pathways (RCPs) of CCSM4 climate change scenario. In general, northeastward range shifts into higher elevation areas of Baekdudaegan Range are notable across all projections. While the extent of habitat suitability was predicted to fluctuate between time frames and scenarios, all projections predicted drastic decrease of habitat suitability, especially across lower elevation areas. Also note the persistence of climatically suitable area, albeit drastically reduced, along the central portion of Baekdudaegan Range. Although slight fluctuations of dispersal limits were predicted across all future projections, no significant expansions or contractions were notable. The map scales and coordinate grids are the same as Figure 2

Consistent with our hypothesis, all future projections predicted a drastic decrease of habitat suitability for *O. koreanus* as well as a general trend of northeastward range shifts into higher elevation area of the Baekdudaegan Range (Figure 4). While the predicted extent of future habitat suitability fluctuated slightly between scenarios and time frames, each future prediction showed drastically decreased habitat suitability compared to that of the current condition. Across all RCPs in both time periods (2050 and 2070), the projected decreases in habitat suitability were characterized by steep declines between the present and the year 2050, with further decreases continuing into the 2070s. The losses of habitat suitability were predicted mostly within the southern and western portions of the current distribution (Figure 4). For both time periods (2050 and 2070) and across all RCPs, areas of high suitability were consistently predicted along the central ridge of the Baekdudaegan Range, representing the eastern edge of current habitat suitability. The models predicted such suitable areas to persist even under the most severe climate change scenario (RCP 8.5 for the year 2070), albeit at greatly reduced extent from the current condition.

### 3.3 Dispersal limits

Our estimation of dispersal limits under the current climate condition was largely matching with the current known distribution of the species. Also, the transition of habitat suitability in central D.P.R. Korea predicted by the current habitat suitability model was generally matching with the northern dispersal limits estimated from the cost distance analysis.

Regarding future projections, all RCPs across both time frames (2050 and 2070) predicted new areas of high suitability to become available in northern D.P.R. Korea, an area characterized by the highest elevation in the Korean Peninsula. While some of these areas fall within the species' dispersal limits under current conditions (Figure 3), estimates under future projections suggest these areas to fall outside the range of the species' dispersal ability, resulting from the contraction of dispersal limits from the current condition (Figure 4). The area of high habitat suitability in southeastern R. Korea predicted under the current conditions mostly remained disconnected from other highly suitable areas across all RCPs. The cost distance analyses on future projections consistently put suitable regions within southeastern R. Korea outside the dispersal limits. Overall, habitat suitability predicted outside of the estimated dispersal limits represents overprediction by the models in areas where the habitat is environmentally suitable but unreachable. It is also notable that while future dispersal limits fluctuated slightly between time periods and scenarios, no great expansion or contraction of dispersal limits was predicted (Figure 4).

### 3.4 Area of suitable and optimal habitats

**FIGURE 5:**
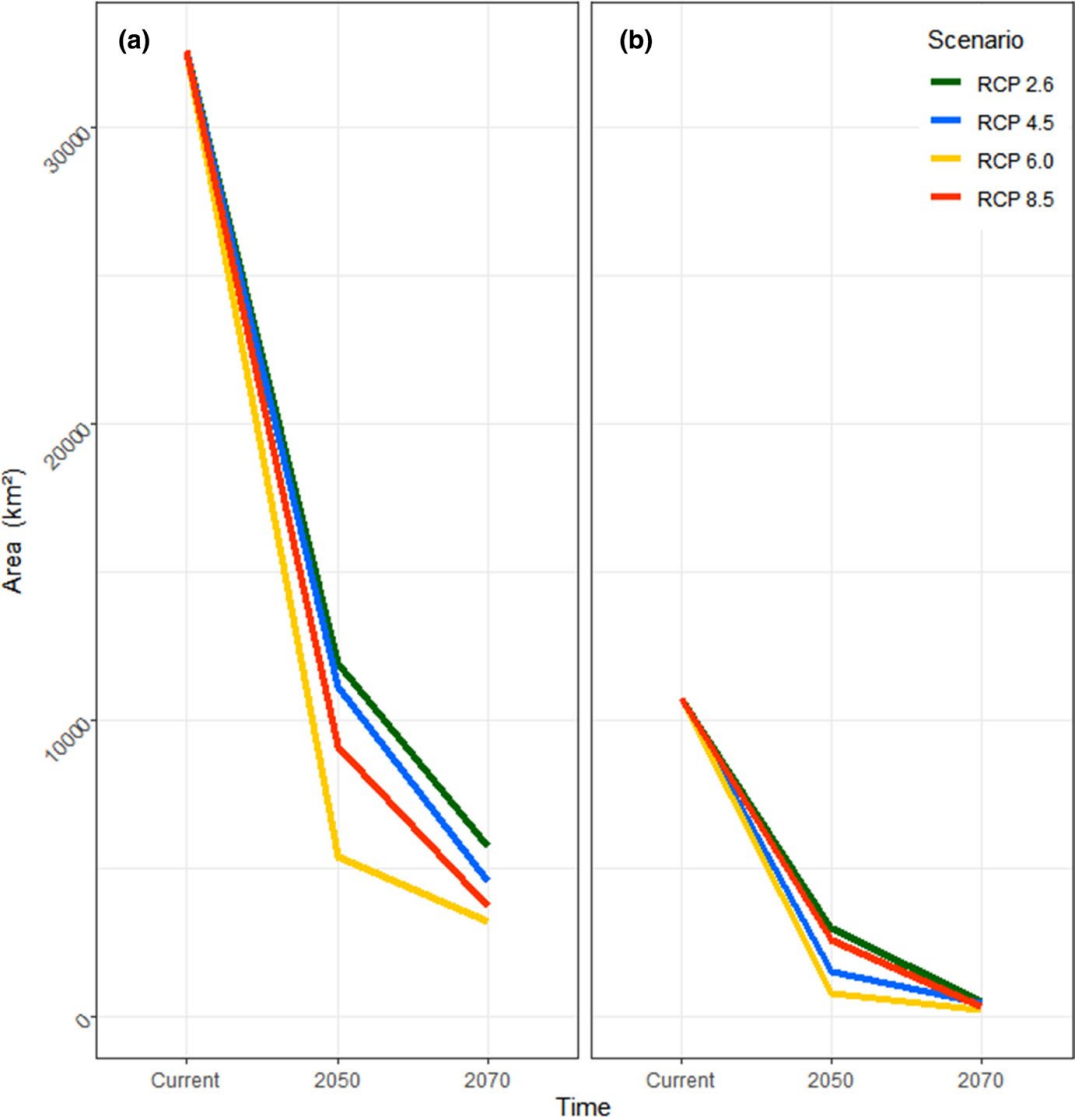
Binary area of presence for *Onychodactylus koreanus* through time, visualized as line plots. (a) Binary area of suitable habitat calculated from maximum training sensitivity plus specificity threshold values. (b) Binary area of optimal habitat calculated from the optimal habitat threshold (= 0.8). Overall, steep declines of both suitable and optimal habitats were predicted as a consequence of projected climate change, with up to 90.1% decrease of suitable area and 98% decrease of optimal area

The total area of current suitable habitat estimated within the dispersal limits was 32,593.1 km^2^ and that of the current optimal habitat was 10,729.4 km^2^. As with continuous habitat suitability, the binary estimation of suitable and optimal habitats was highlighted by a drastic contraction. The models predicted sharp decreases of both suitable and optimal habitats between the present and 2050, with further decreases predicted for 2070 (Figure 5). Depending on years and RCPs, our models predicted as much as 90.1% of decrease for the suitable area (under RCP 6.0 in 2070) and 98% of decrease for the optimal area (under RCP 6.0 in 2070) compared to the current conditions (Table 1; see Appendices E and F for binary grids of suitable and optimal habitats). Across all RCPs in both time periods (2050 and 2070), the elevational ranges of future suitable and optimal areas were significantly different from elevational ranges of the current conditions (Mann–Whitney *U* tests; *p* < .001; Table 2). In general, the elevational shifts were toward higher elevation regions of the Baekdudaegan Range.

**TABLE 1:** The total area of suitable and optimal habitats calculated for 2050 and 2070, along with percent decrease from the current area of suitable (32,593.1 km^2^) and optimal (10,729.4 km^2^) habitats

Scenario	2050				2070			
	Suitable area (km^2^)	Percent decrease (%)	Optimal area (km^2^)	Percent decrease (%)	Suitable area (km^2^)	Percent decrease (%)	Optimal area (km^2^)	Percent decrease (%)
RCP 2.6	11,912.3	63.5	2,961	72.4	5,727.7	82.4	482.7	95.5
RCP 4.5	11,118.6	65.9	1,524.3	85.8	4,568	86	446	95.9
RCP 6.0	5,403.8	83.4	796.3	92.6	3,217.7	90.1	213.3	98
RCP 8.5	9,129.6	72	2,591	75.9	3,718.6	88.6	321.2	97

Note

Optimal habitats (suitability >0.8) for *Onychodactylus koreanus* are predicted to effectively disappear from the Korean Peninsula by 2070. The total area of suitable habitats is also similarly characterized by steep decline over time.

**TABLE 2:** The results of Mann–Whitney *U* tests conducted between elevational ranges of current suitable and optimal areas and elevational ranges of each RCP scenario

Year	Scenario	Suitable area		Optimal area	
		*W*	*p‐*value	*W*	*p‐*value
2050	RCP 2.6	315,304,109	<.001	27,161,299	<.001
	RCP 4.5	18,974,546	<.001	14,881,389	<.001
	RCP 6.0	116,685,642	<.001	5,648,708	<.001
	RCP 8.5	230,253,203	<.001	24,237,800	<.001
2070	RCP 2.6	136,470,183	<.001	3,126,799	<.001
	RCP 4.5	114,520,671	<.001	3,639,348	<.001
	RCP 6.0	84,823,384	<.001	1,924,267	<.001
	RCP 8.5	100,279,998	<.001	3,279,824	<.001

## 4 DISCUSSION

In agreement with our hypothesis, the models predicted drastic decreases of future habitat suitability for *Onychodactylus koreanus*, leading to an almost complete loss of both suitable (~90.1%) and optimal (~98%) habitats by the year 2070 under the RCP 6.0 scenario. In addition, the models predicted significant range shifts in the future, generally characterized by a northeastward directional shift toward higher elevation areas of the Baekdudaegan Range and the loss of habitat suitability across the western and southern portions of the current suitable habitats. This general pattern is largely similar to previous modeling‐based studies on other species of salamanders, including the Chinese Giant Salamander (*Andrias davidianus*; Zhang, Mammola, et al., 2020; Zhang, Dong, et al., 2020), the Korean Crevice Salamander (*Karsenia koreana*; Borzée, Andersen, et al., 2019), and the Lorestan Mountain Newt (*Neurergus kaiseri*; Ashrafzadeh et al., 2019).

Increased annual mean temperature and increased fluctuations in precipitation are likely driving the loss of suitable habitat and range shifts predicted in our models. Such environmental changes triggered by rapid climate change are likely to cause habitat degradation by altering a suite of important habitat requirements for the species. For example, the species is highly dependent on low‐temperature mountain forests receiving abundant precipitation (Poyarkov et al., 2012; Lee & Park, 2016). However, future climate change scenarios for R. Korea indicate an increased severity of droughts (Kim et al., 2013), as well as an increase in extreme precipitation events (Sung et al., 2018). Therefore, increasing fluctuations in temperature and precipitation are likely to create habitat conditions unsuitable for the species by causing desiccation and flooding of habitats and affecting the survival of both larvae and adults. In addition, the breeding environment of the species is strongly associated with unpolluted, low‐temperature mountain streams with high levels of dissolved oxygen (Hong, 2017; Jung, 2020; Lee & Park, 2016; Park, 2005), and therefore, larvae of the species are highly dependent on these requirements (Hong, 2017; Jung, 2020; Kuzmin, 1995). The amount of dissolved oxygen decreases with increasing temperature in the aquatic environments (Ficklin et al., 2013; Matear et al., 2000), and such decreases in dissolved oxygen can negatively impact amphibian communities (Bickford et al., 2010). Therefore, warming climate can result in degradation of the mountain stream environments by reducing the amount of dissolved oxygen. The combination of these factors is particularly negative to the larvae considering the extended aquatic larval stage of the species (2–3 years; Lee & Park, 2016). Furthermore, climate change can also cause climatic disturbances and subsequent degradation of subterranean environments used by the species for reproduction (Mammola et al., 2018, 2019; Park, 2005; Zhang, Mammola, et al., 2020).

In addition, complex interactions between climate change and other factors not accounted for in our models are likely to have negative impacts on the population dynamics of the species. For example, while our models assumed constancy of land‐cover variables through time due to the lack of applicable future scenarios, forest environments will most likely be affected by climate change (Choi et al., 2011; Joshi et al., 2012). In R. Korea, a drastic contraction of the forest volume has been predicted under future climate conditions (Kwak et al., 2012). In addition, cool temperate forests that are the principal habitats of *O. koreanus* have been predicted to be replaced by subtropical vegetation by 2100 due to climate change (Choi et al., 2011). The specific impacts of replacement and shift of vegetation on population dynamics are difficult to assess at present, but it will most likely lead to serious habitat degradation and population decline due to the loss of key habitats (Cushman, 2006).

Finer scale environmental variables that are important in determining habitat suitability, such as soil pH, are also likely to change with climate warming. Acidic soils negatively affect amphibian communities through disturbances in osmoregulation, growth, and respiration (Sugalski & Claussen, 1997; Wyman & Jancola, 1992), and the habitat requirements of *O. koreanus* reflect avoidance of environments with low soil pH (Hong, 2017; Jung, 2020). Soil acidification can be exacerbated by altered precipitation patterns caused by climate change (Rengel, 2011), resulting in decreased habitat suitability. While microhabitat features and plasticity can provide buffering effects to climate change (Huey et al., 2018; Scheffers et al., 2014), habitat degradation at multiple levels is still likely to put severe pressure on the species in the future.

Other anthropogenic stressors are additional threats to the species. Widespread threats such as deforestation, pollution, road construction, and conversion of landscapes to agriculture put a strong pressure on amphibian populations by hampering dispersal, reproduction, foraging, and other behaviors associated with species survival (Beebee & Griffiths, 2005; Lee & Miller‐Rushing, 2014; Shin, Jeong, et al., 2020). Such development of the habitat is ongoing and causing widespread environmental degradation across the Korean Peninsula (Lee & Miller‐Rushing, 2014; Park et al., 2014). This is particularly more problematic for R. Korea, where the rate of development is high (Borzée, 2021; Borzée et al., 2019; Lee & Miller‐Rushing, 2014) and where the majority of suitable habitats for *O. koreanus* is located. The rate of development on the Korean Peninsula in the future and its impact on the future habitat suitability of *O. koreanus* are difficult to predict. However, if the current rates were to continue or even increase, our predictions of future habitat suitability could only become worse for the species. Thus, there are multiple complex environmental factors acting in concert with climate change in determining future habitat suitability of *O. koreanus*. Taking this negative pressure into account is of primary importance as such changes are imminent or ongoing and pose serious conservation concerns for the species.

We do acknowledge some limitations to our models. For example, while we employed cost distance analyses to delimit the dispersal limits of *O. koreanus*, these dispersal limits do not translate into accurate range boundaries for the species. Nevertheless, such estimates are still useful because: (a) the dispersal ability of *O. koreanus* is unknown, (b) the distribution and species‐level identification of *Onychodactylus* in D.P.R. Korea are uncertain, and (c) conducting fieldwork in D.P.R. Korea to resolve such uncertainties is difficult at present. Therefore, we consider using approximate range boundaries to be more beneficial than not specifying range boundaries at all. To partly overcome such uncertainties, additional studies on the movement ecology of *O. koreanus* are needed to gain better understanding of the species' dispersal ability, and thereby improving SDMs.

Ecological and especially physiological investigations including critical thermal maximum (CT_max_) may provide valuable insights into the effects of warming climate on the species' survival (Duarte et al., 2011). Due to the lack of such knowledge for the species at present, our models cannot mechanistically account for the role of climate change in determining the future distribution of *O. koreanus*. Therefore, the prospect of obtaining mechanistic SDMs is vastly more difficult than correlative SDMs, and the latter method remains the only feasible method of modeling.

While our models suggest a drastic decrease of future habitat suitability, there are opportunities for conservation. Across our future projections, a persistence of suitable habitats was predicted across the central portion of the Baekdudaegan Range along the east coast of the Korean Peninsula. This region likely represents a climatic refugium and may warrant recognition as an area of conservation priority for the species in the future. There are several protected areas in this region including Seoraksan, Odaesan, Taebaeksan, and Geumgangsan National Parks. However, the combined area of these national parks represents only a small fraction of the total area of the Baekdudaegan Range, and therefore, the established parks are currently insufficient to protect the habitat and species from climate change and environmental degradation. Thus, the designation of additional protected areas in the future is needed to better protect suitable habitats for the species.

In addition, our study has implications for the legal protection and threat status listing of *O. koreanus*. In the R. Korea, *O. koreanus* is currently listed as Least Concern (LC) under the national Red List (NIBR, 2019), while it is also listed as LC by the IUCN Red List of Threatened Species (Kuzmin et al., 2004). However, our results suggest that the conservation status of the species will require close attention. With warming climate and continuing habitat degradation, the species may warrant protection under a threatened category at the national level again in the future. In addition, the IUCN listing (Kuzmin et al., 2004) also needs to be updated as it is considered out of date and in need of update 10 years after release. For D.P.R. Korea, identifying the conservation needs of the species remains difficult due to the general paucity of data. The environments of D.P.R. Korea have experienced significant degradation due to the past deforestation and habitat fragmentation (Kang & Choi, 2014; Lee & Miller‐Rushing, 2014), likely having negative impacts on the populations of *O. koreanus*. However, the population status of *O. koreanus* is unknown for the nation, and while available literature on the herpetofauna of D.P.R. Korea suggest local abundance (Kim & Han, 2009; Won, 1971), population trends are difficult to assess without additional data, especially in light of uncertain species‐level identification.

With additional threats of climate change and conservation needs identified, we highlight the foremost importance of gathering sufficient ecological knowledge on the species to implement effective conservation actions in the future. At present, only very limited ecological information is available, and there are no reliable records of population size. Successful conservation and management actions are ideally backed by sufficient ecological information on the target species (Iskandar & Erdelen, 2006). Therefore, we urge additional ecological studies and population monitoring on the species to be conducted and, ideally, the establishment of long‐term population monitoring programs. Such programs can be implemented in the form of regular surveys conducted by researchers and can also benefit from the participation of citizen scientists (Borzée et al., 2018; Borzée, Baek, et al., 2019; Deutsch et al., 2017).

Our results are also applicable to other species of *Onychodactylus*. While we used *O. koreanus* as an example, the potential negative impacts of climate change on future habitat suitability are not unique to the species. All ten known species of *Onychodactylus* are lungless and share largely similar environmental requirements for survival (Poyarkov et al., 2012; Yoshikawa & Matsui, 2013, 2014; Yoshikawa et al., 2013). Therefore, all of these species are likely to face similar challenges under rapid climate change (Pyron, 2018). *Onychodactylus* represents an ancient lineage of salamanders (Frost et al., 2006; Pyron & Wiens, 2011), and assessing the potential impacts of climate change and other anthropogenic pressures on the genus is required to better understand and conserve the unique evolutionary diversity of this lineage.

## CONFLICT OF INTEREST

The authors declare no conflict of interest.

## AUTHOR CONTRIBUTIONS

**Yucheol Shin:** Data curation (equal); formal analysis (equal); investigation (equal); methodology (equal); software (equal); validation (equal); visualization (equal); writing‐original draft (equal); writing‐review & editing (equal). **Mi‐Sook Min:** Conceptualization (equal); funding acquisition (equal); data curation (equal); project administration (equal); validation (equal); visualization (equal); writing‐review & editing (equal). **Amaël Borzée:** Conceptualization (equal); funding acquisition (equal); investigation (equal); methodology (equal); project administration (equal); resources (equal); software (equal); supervision (equal); validation (equal); visualization (equal); writing‐review & editing (equal).

## Data Availability

The data used in this study are available from the Mendeley Data repository (https://doi.org/10.17632/yc3nw4d9f2.2; Shin, Min, et al., 2021) and the appendices accompanying this manuscript.

## APPENDIX A

The original and reclassified values of landscape variables used to create cost rasters. We reclassified the original raster values based on our knowledge of the species. The “thresholds” specified for reclassification of habitat suitability output models are based on maximum training sensitivity plus specificity thresholds computed in MaxEnt runs. The cost rasters resulting from these reclassified rasters were used as inputs for cost distance analyses to estimate dispersal limits of *Onychodactylus koreanus* under each climate change scenario (see text for methods).

**Table:**

Variables	Original raster values	Reclassified cost intervals
Barren landscape cover	0 – 10	1
	10–20	5
	20–30	10
	30–40	30
	40–60	50
	60–100	1,000,000
Human footprint	4–10	1
	10–15	5
	15–20	10
	20–30	1,000
	30–50	1,000,000
Cultivated landscape cover	0–10	1
	10–20	5
	20–40	10
	40–60	50
	60–80	100
	80–100	1,000,000
Elevation	−44 to 0	1,000,000
	0–10	500,000
	10–100	100
	100–200	10
	200–800	1
	800–1,000	10
	1,000–1,344	50
	1,344–2,460	1,000,000
Open water	0 (absent)	1
	1 (present)	1,000,000
Forest cover	0–10	1,000,000
	10–20	500,000
	20–30	100,000
	30–40	10,000
	40–50	1,000
	50–60	100
	60–70	10
	70–85	5
	85–100	1
Urban landscape cover	0 (nonurban)	1
	1 (urban)	1,000,000
Current habitat suitability (threshold = 0.5175)	0–0.5175	1,000,000
	0.5175–0.6	50
	0.6–0.8	10
	0.8–1.0	1
2050 RCP 2.6 (threshold = 0.4804)	0–0.4804	1,000,000
	0.4804–0.6	50
	0.6–0.8	10
	0.8–1.0	1
2050 RCP 4.5 (threshold = 0.5013)	0–0.5013	1,000,000
	0.5013–0.6	50
	0.6–0.8	10
	0.8–1.0	1
2050 RCP 6.0 (threshold = 0.4942)	0–0.4942	1,000,000
	0.4942–0.6	50
	0.6–0.8	10
	0.8–1.0	1
2050 RCP 8.5 (threshold = 0.506)	0–0.506	1,000,000
	0.506–0.6	50
	0.6–0.8	10
	0.8–1.0	1
2070 RCP 2.6 (threshold = 0.4729)	0–0.4729	1,000,000
	0.4729–0.6	50
	0.6–0.8	10
	0.8–1.0	1
2070 RCP 4.5 (threshold = 0.4744)	0–0.4744	1,000,000
	0.4744–0.6	50
	0.6–0.8	10
	0.8–0.93821	1
2070 RCP 6.0 (threshold = 0.4926)	0–0.4926	1,000,000
	0.4926–0.6	50
	0.6–0.8	10
	0.8–1.0	1
2070 RCP 8.5 (threshold = 0.5473)	0–0.5473	1,000,000
	0.5473–0.6	50
	0.6–0.8	10
	0.8–1.0	1

## APPENDIX B

Specific threshold values used for binary area calculations from each climate change model of *Onychodactylus koreanus* across the Korean Peninsula. The models are based on 187 georeferenced occurrence records. The suitable habitat thresholds are based on the maximum training sensitivity plus specificity threshold computed in MaxEnt runs. The optimal habitat threshold is defined following Mothes et al. (2020).

**Table:**

Scenario	Suitable habitat threshold	Optimal habitat threshold
Current	0.5175	0.8
2050 RCP 2.6	0.4804	0.8
2050 RCP 4.5	0.5013	0.8
2050 RCP 6.0	0.4942	0.8
2050 RCP 8.5	0.506	0.8
2070 RCP 2.6	0.4929	0.8
2070 RCP 4.5	0.4744	0.8
2070 RCP 6.0	0.4926	0.8
2070 RCP 8.5	0.5473	0.8

## APPENDIX C

Percent contribution and permutation importance for each environmental variable used to estimate current habitat suitability of *Onychodactylus koreanus*. Based on 187 data points collected between 1911 and 2020 on the Korean Peninsula.

**Table:**

Variable	Percent contribution	Permutation importance
Slope	41.4	33.8
Annual mean temperature (bio1)	17.6	0.1
Precipitation of driest months (bio14)	13.4	31.1
Precipitation of wettest months (bio13)	9	21.5
Mixed/other trees cover	5.6	1.9
Isothermality (bio3)	4.9	0
Temperature seasonality (bio4)	4.1	3.7
Needleleaf forest cover	3.9	8

## APPENDIX D

Response curves of *Onychodactylus koreanus* to environmental variables used for model prediction. The plots are in order of highest contribution from top to bottom and left to right. See Appendix C for contribution value of each variable. Based on 187 data points collected between 1911 and 2020 on the Korean Peninsula.

## APPENDIX E

Binary presence grids generated from output models based on suitable habitat thresholds. The map scales and coordinate grids are the same as Figure 2.

## APPENDIX F

Binary presence grids generated from output models based on the optimal habitat threshold. The map scales and coordinate grids are the same as Figure 2.
